# Point of care lung ultrasound in COVID-19: hype or hope?

**DOI:** 10.1259/bjro.20200027

**Published:** 2020-08-28

**Authors:** Abdulrahman M. Alfuraih

**Affiliations:** 1 Department of Radiology and Medical Imaging, College of Applied Medical Sciences, Prince Sattam bin Abdulaziz University, Kharj, Saudi Arabia

## Abstract

The COVID-19 coronavirus pandemic has critically struck the world economy and healthcare systems. The highly contagious virus spreads rapidly and can result in potentially life-threatening acute respiratory distress. The current established test for diagnosing COVID-19 is using the RT-PCR laboratory test. However, the test requires specialized laboratories and testing kits. Recent reports also showed high false-negative rates. Experts recognize the urgent need to develop a rapid point of care diagnostic tests. Ultrasonography is a widely established safe diagnostic imaging test for detecting various lung abnormalities. Recent publications from China and Italy provided limited evidence on its usefulness for diagnosing COVID-19 in emergency departments earlier than RT-PCR. Ultrasound is sensitive to pleural and subpleural abnormalities, which suggests a great potential diagnostic role given the predilection for COVID-19 in peripheral subpleural regions.This paper reviews the current evidence and discusses the problems with specificity and scoring.

After the initial outbreak of the SARS-CoV-2 virus in Wuhan, the world got into a state of turmoil with total cases exceeding 15 million as of July 2020. It is an extremely contagious virus, which could potentially cause acute respiratory distress and potentially organ failure in advanced stages.^1^

COVID-19 is diagnosed by reverse-transcription polymerase chain reaction (RT-PCR), which was criticized after reports of poor diagnostic sensitivity.^2,3^ In contrast, alternative diagnostic tests such as chest CT has not been recommended by international and local guidelines due to several reasons, including poor specificity and radiation risks.^4^ Lung ultrasonography (LU) is an established technique for evaluating lung pathologies. It is sensitive to abnormalities in the pleura and subpleural spaces. The initial evidence on COVID-19 suggesting peripheral lung involvement at the terminal alveoli, which alludes a significant role for LU in diagnosis and management. This paper presents an up-to-date review of the LU evidence in COVID-19 and discusses the pitfalls and future perspectives.

## Lung ultrasound in COVID-19

There have been several publications describing the role of LU in COVID-19.^5–16^ The publications were divided between simple case-studies, letters of opinion and commentaries discussing the potential usefulness of LU. Despite a few useful descriptions of how LU can be included in the COVID-19 diagnostic workup and management, they present inconclusive results and apparent lack of thoroughness. The paragraphs below discuss the overall findings.

The first case report was of a 57-year-old male presenting to the ER with fever, cough, general weakness, and headache. The LU findings showed bilateral irregular pleural line with minimal consolidations and thick multiple B-lines in the anterior and posterior hemithorax. As a control case, a suspected patient (later confirmed negative) with typical COVID-19 symptoms exhibited normal lung pleura with A-lines on LU. Notably, the scan was performed by two operators using a handheld device, one scanning and the other holding the tablet (freezing and storing images). This practice was recommended by them to mitigate the risks of infection. The second case study^6^ was of a female heath practitioner in her sixties with confirmed COVID-19 that developed a cough and sore throat followed by dyspnea. LU demonstrated multiple B-lines, consolidation and thickened pleural line. Both of these cases can be considered moderate in terms of severity (both febrile with low oxygen saturation). The third, and most interesting, case study^14^ was of pregnant females with positive lung ultrasound findings suggesting COVID-19 which had an early negative PCR result. The findings included bilateral thick B-lines in posterior lung regions, which correlated perfectly with the CT results. She later tested positive on RT-PCR. This report demonstrates the early detection capability of LU in cases with false-negative RT-PCR results. It also further supports Ai et al.^3^ recommendations regarding the importance of lung imaging considering the high sensitivity prior to positive RT-PCR results.

Several publications included descriptive case series. Huang et al were the first to publish a case series on 20 patients of non-critical patients in the emergency department.^11^ Despite not being peer-reviewed, their report described lesions located bilaterally in the posterior inferior lung regions. They were characterized by fixed confluent B-lines (waterfall sign), diffused B-lines (white lung sign), irregular and thickened pleural line, and consolidation with air bronchograms. Additionally, they reported localized pleural effusion in a few cases (exact number not mentioned), which is seldom found in viral pneumonia. They highlighted that these are characteristic findings different from bacterial pneumonia or pulmonary edema, where B-lines are mobile and focal. We argue otherwise as explained below.

In the emergency settings, Peng et al published a brief letter explaining the ultrasonographic features of COVID-19 in 20 cases.^9^ They reported the same findings of Huang et al except for finding focal B-lines. An Italian group of researchers published a letter on 12 patients presenting to the ER.^10^ In agreement with the above reports, they found diffused B-lines in all patients and posterior consolidation in three patients only. They also mentioned a good correlation between CT and LU.

All reports above omitted reporting the severity of the cases. This is vital information as it can be postulated that LU features vary depending on the disease development. Additionally, none of the case series examined the usefulness of handheld ultrasound devices. With regard to LU scanning techniques, there is a clear discrepancy in the number of regions screened. Some studies opted for the limited four-zone testing, while others employed the 12- and 14-zone protocols. Soldati et al^12^ proposed an unvalidated protocol to perform LU in COVID-19 patients. They recommended scanning patients in a sitting position (when possible) using convex or linear transducers operating low mechanical index settings with a single focus point on the pleura. However, their recommended 14-zone scanning protocol is cumbersome and has not been described before. It extends the 12-zone protocol by adding two extra zones at the back of the chest. In our experience, the lung is mostly obscured at the back by the scapula, and the 12-zone is a sufficient protocol to thoroughly scan the lung even in ICU settings where the scanning time should be short. It was also the employed protocol in multiple publications^5,9,11,13^ and endorsed by multiple societies.^17^

Vetrugno et al described quite positively their Italian experience with LU in COVID-19.^13^ They noted the application of LU helped in decreasing the rates of chest x-rays and CT scans, which contributed to making care delivery and management more efficient. They also described a total scanning time of less than 5 min, which we believe underestimates the actual whole time especially with dressing, positioning and documentation. Despite the promising results of accuracy, efficiency, safety, reproducibility, and point of care use of LU, it has several drawbacks. For example, LU is limited to visualizing the pleural surface and cannot detect deep lung lesions. However, this may not be an issue considering the predilection of COVID-19 in peripheral subpleural regions. A second limitation is a need for experienced users to operate the ultrasound device. Practitioners should also be aware that LU, like CT, does not exclude COVID-19 in subjects with no pulmonary complications.

## The specificity problem

The LU features of COVID-19 include irregular pleural line, confluent B-lines, and consolidations in more severe cases. Two studies described pleural effusion, which is seldom found in viral pneumonia. The problem is that none of these signs are pathognomonic for COVID-19 as they merely describe the density state of the lung surface. We should be cautious when calling these signs characteristics amidst hospitals flooding with COVID-19 as the pre-test likelihood for positive findings in suspected COVID-19 is currently high.

Few researchers argue the presence of a typical LU sign coined as the ‘light beam artifact’ characterized by a vertical echoic broadband-like artifact, similar to the white lung sign, which comes in and out of the frame with respiration.^18,19^ This feature corresponds to acute ground glass alteration which are prevalent in COVID-19 pneumonia. It may be a specific sign especially when imaged in young patients with no history of lung diseases. Nevertheless, the described features bring some hope. They can be considered significantly different to signs of pulmonary fibrosis and interstitial pneumonia caused by other viruses where a diffused B-lines with no spared areas.

## Image scoring

There is currently no validated image scoring system for diagnosing COVID-19 on LU. Soldati et al^12^ proposed a scoring criterion for LU images. It scores each zone in the 12-zone LU protocol from 0 (normal) to 3 (severe) based on the LU lung involvement patterns relating to pleural lines, vertical artifacts and consolidations. This scoring system was also recently proposed by Manivel et al^20^ but with minor modifications in each category definition. This semiquantitative scoring system is a promising attempt to standardize the reporting of COVID-19 scans. However, it was developed based on the sole observations of the research team of Soldati et al.^12^ Hence, the criterion and content validity must be established as well as the inter- and intra-reader reproducibility.

It can be difficult to appreciate or count the number of B-lines especially as minute changes can significantly influence the image. Hence, we recommend obtaining the best possible image for each zone then ‘freezing’ to count the lines for scoring. A best practice approach is to acquire a clip and still images of each zone for post scanning documentation, inter-reader deliberation and medicolegal purposes.

## Future perspectives

To date, all results provided positive outcomes on the usefulness of LU in COVID-19. However, the presented evidence thus far is considered weak. None of the studies included a control group or reported their findings longitudinally. Moreover, the sample sizes were extremely small, impeding results generalization. Several international societies, such as the British Medical Ultrasound Society, the intensive care society, and the WFUMB are calling for more research on LU in COVID 19. Future research should focus on validating the LU signs and patterns based on the proposed scoring system to suggest optimum cut-off scores.

In our opinion, LU in COVID-19 can have elevated rates of false-positives due to the overlapping features with other viral pneumonia. LU findings should never be interpreted without consideration of the clinical context (*e.g.,* symptoms, decision to supplement oxygen). For instance, it can be used to check lung aeration before and after non-invasive ventilation. This may indicate that there is more hope for LU in COVID-19 as a bed-side-management tool for confirmed cases, potentially saving them from re-exposure to ionizing radiation. However, in screening tests, it is better to have a high sensitivity than high specificity. As a false-negative case infected with COVID-19 can be discharged and mingle back in society, eventually infecting numerous new cases.

The novel coronavirus unprecedented healthcare crisis indisputably calls for point of care screening solutions that can deliver rapid and sensitive results. The previous and recent evidence suggests that LU could be the called-for solution to diagnose positive cases and grade their severity. Modern handheld ultrasound devices can be particularly useful considering their relatively cheap prices and ease of disinfection without significant compromises on quality. The future potential for a radiation-free and relatively cheap bed-side-tool carries hope.
